# Popular Medical Lectures

**Published:** 1904-04-23

**Authors:** 


### Popular Medical Lectures.

The medical profession is not, and we hope it never will be, chary of spreading any knowledge which by preventing disease will both avoid individual suffering and promote national well-being. It is, indeed, the legitimate boast of the profession that both in private duties and official responsibilities it prosecutes the public welfare irrespective of selfish and individual claims. What knowledge is gained is not kept secret for the sake of private ends, but is freely offered and utilised in the public interest. It may, however, be questioned whether this highly laudable motive does not at times lead to practices which are to the advantage neither of the public nor the profession. This, we think, may be seen in the increasing development of the habit of delivering lectures on questions of health and disease to popular audiences by medical men. That there are certain subjects, some having a scientific interest, others an immediate practical value, capable of presentation to popular audiences, need not be denied. The general problems of physiology, instruction in "first aid," the social and national relationships of tuberculosis may be quoted as examples. In none of these is the province and responsibility of the individual practitioner invaded, and each may be treated on broad lines and without any serious difficulty in the adjustment of principles to the details of practical application. It is decidedly different when entrance is made into regions where the treatment of individuals is concerned, for here knowledge and the exercise of judgment, only to be obtained by training and experience, are essential. Is it for example to be expected that a popular audience is likely to benefit from a course of lectures on the subject of infant feeding? The probability is that most members of the audience will retain some general rule—or perhaps an exception to the rule—and will insist on applying this, unmindful of modifying circumstances, over the entire field of influence under their control. Advice regarding the feeding of any individual infant is eminently a question for the family medical adviser, who alone is in a position to appreciate all the circumstances. Against certain pernicious dietetic practices more or less common among the poorer classes, a warning may well be given, as is the case in some of the large towns, by means of a leaflet handed to every person who registers the birth of a child. But beyond this there is, it seems to us, little or no room for public instruction in this matter, and attempts at such instruction are not unlikely to produce a vainglorious self-confidence which regards itself as entirely independent of the family medical adviser. If the lectures are merely a repetition of principles and methods generally recognised in the profession, what need exists for their public exposition, and why is one member of the profession rather than another to proclaim himself as specially competent to act as an evangelist and apostle? It has also to be remembered that even on such a subject as infant feeding there are points—and those not of mere remote or theoretical interest—on which men of equal competence and experience differ. If one authority may expound his views to popular audiences, obviously equal freedom must be allowed to others. Indeed, there can be no possibility of preventing, at least by any consistent policy, any medical man, who cares to do so, from lecturing in public on any subject which may possess popular interest even though in practice the principles he announces require for their safe application the directions of an expert medical adviser. Even already the private practitioner occasionally finds his procedure challenged on the ground that it is not in harmony with Dr. So-and-So's lectures. The conclusion would appear to be that upon questions of public health, upon the immediate treatment of emergencies, there is room for public instruction by members of the profession, and though private practitioners may in particular circumstances take part in this, it were best as far as possible to leave it in the hands of those engaged in the public service. Once this policy is departed from there is the opportunity for serious evils, and neither purity of motive nor elevated position can absolve those who transgress traditions and practices which are not the less in the public interest because they have enjoyed a long-established professional sanction.

